# Immune-dominated cellular heterogeneity and stromal plasticity in keloid infiltrating and hypercellular zones revealed by single-cell RNA sequencing

**DOI:** 10.3389/fimmu.2026.1873878

**Published:** 2026-06-26

**Authors:** Yuchen Cao, Li Duan, Dongxian Lin, Yue Liu, Mingzi Yang, Shunbing Lu, Yonghao Liu, Yuanyuan Xu, Lianzhao Wang

**Affiliations:** 1 Department of Cicatrix Minimally Invasive Treatment Center, Plastic Surgery Hospital, Chinese Academy of Medical Sciences and Peking Union Medical College, Beijing, China; 2 Plastic Surgery Hospital, Chinese Academy of Medical Sciences and Peking Union Medical College, Beijing, China; 3 Renmin Hospital of Wuhan University, Wuhan University, Wuhan, China

**Keywords:** hypercellular zone, immune microenvironment, infiltrating zone, keloid, single-cell sequencing

## Abstract

**Background:**

Keloids are aggressive fibroproliferative disorders characterized by a sclerotic core and an actively invading margin, yet the cellular and molecular basis of this spatial heterogeneity remains poorly understood. The roles of immune-stromal crosstalk in driving peripheral invasion have not been systematically dissected.

**Methods:**

Paired infiltrating and hypercellular zones were collected from anterior chest keloids of four patients. Single−cell RNA sequencing was performed on 128,678 cells after rigorous quality control. Unbiased clustering, differential gene expression analysis, pseudotime trajectory reconstruction, and cell-cell interaction profiling were applied to compare the landscapes of cell composition, function, and differentiation/development trajectories between the two zones.

**Results:**

The hypercellular zone was dominated by extracellular matrix−producing myofibroblasts, whereas the infiltrating zone was enriched in mononuclear phagocytes and endothelial cells, exhibiting a loose collagen architecture permissive for cellular invasion. Infiltrating zone fibroblasts adopted an immunomodulatory, inflammatory cancer−associated fibroblast−like phenotype, while macrophages displayed a type I interferon signature and immunoglobulin−mediated activation, indicating a chronic inflammatory state with features reminiscent of certain autoimmune conditions. Langerhans cells followed a three−stage developmental trajectory from a stress−responsive to a terminal NF−κB−driven effector state, orchestrating neutrophil and Th17 cell recruitment via chemokine and cytokine networks, with significant enrichment of the IL−17 signaling pathway. Endothelial cells at the margin underwent endothelial−to−mesenchymal transition, and Schwann cells exhibited phenotypic plasticity, mirroring aggressive tissue remodeling.

**Conclusions:**

In the context of this spatially resolved analysis, this study redefines the keloid margin as an immunology−dominated niche characterized by profound cellular and spatial heterogeneity that is associated with features that could drive peripheral invasion. These findings suggest a shift of the paradigm of keloid pathogenesis from a fibroblast−centric model to an immune−driven integrated landscape in this cohort and provide a theoretical foundation for keloid precision therapies and zone−specific biomarkers for treatment response and disease monitoring.

## 1 Introduction

Keloid, an abnormal and aggressive form of cutaneous fibroproliferative disorder, is characterized by excessive fibroblast proliferation and pathological deposition of extracellular matrix (ECM) beyond the original wound margins (1, 2). Despite the implementation of various therapeutic interventions, including surgical excision, radiotherapy, and intralesional corticosteroid injections, recurrence rates remain high, thereby posing significant clinical challenges (3, 4). A more profound comprehension of its pathogenesis, particularly the interaction between fibroblasts and immune cells within the keloid microenvironment, is imperative for the development of more effective and targeted therapies (5).

Single-cell RNA sequencing (scRNA-seq) offers transformative advantages for advancing our understanding of keloid pathogenesis (6, 7). Traditional bulk RNA sequencing analyzes the average gene expression of entire tissue samples, masking the heterogeneity within the complex keloid microenvironment. In contrast, scRNA-seq enables the unbiased, high-resolution dissection of this landscape at the individual cell level (8, 9). It allows for the comprehensive identification and molecular characterization of all cellular constituents (10, 11).

**Figure 1:**
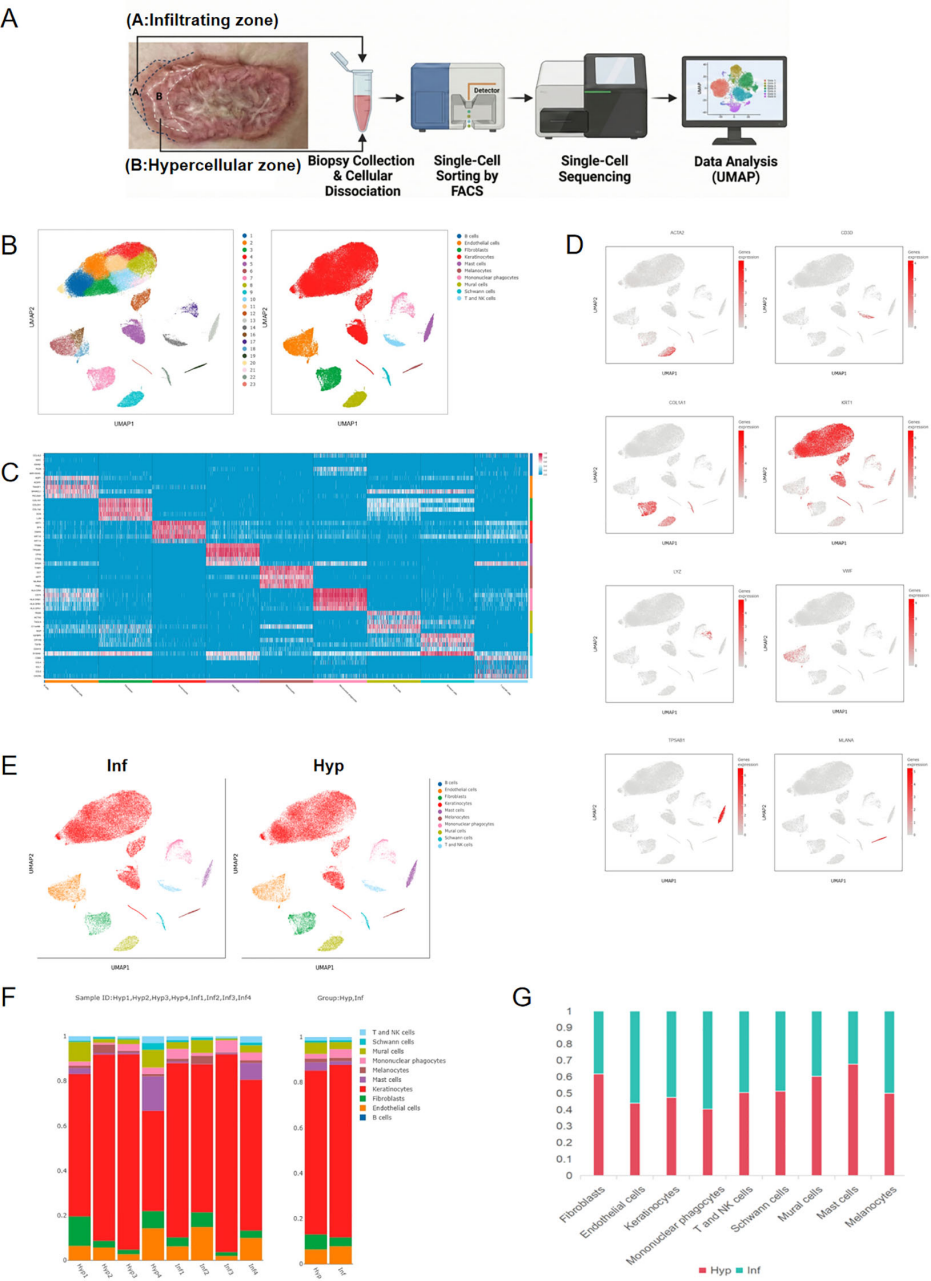
Overall differences of scRNA-seq results between keilod infiltrating and hypercellular zones. **(A)** Workflow diagram of collecting the specimens from different zones in keloid and single-cell sequencing. **(B)** The UMAP plot of cells unbiased clustering results and major categories clustering results based on marker genes. Each color and number coded to indicate the associated cell types. **(C)** Cluster heatmap of DEGs of all major cell types. **(D)** Feature plots depicting the normalized expression profiles of marker genes in cell clusters. Expression levels (gray to red gradient) are visualized on UMAP plot. **(E)** The distribution of major cells derived from two groups samples in UMAP plot. **(F)** Proportion plot of cell types composition between two groups and eight samples. **(G)** The distribution of all cell types derived from two groups samples.

Building upon our preliminary clinical observations and previous clinical and basic research on keloids (12, 13), we found that different zones of keloids exhibit significant variations in sensitivity and efficacy to the treatment of topical injection of 5-fluorouracil and triamcinolone (3, 14, 15). Consequently, to achieve more precise treatment, we propose classifying the zones surrounding the aging core region of keloids into a hypercellular zone (Hyp) and an infiltrating zone (Inf) (13, 16). The hypercellular zone constitutes the core area of keloid proliferation, characterized by marked elevation, a dark red coloration, and considerable firmness (**Figure 1A**). The infiltrating zone, conversely, represents the junction between the keloid and normal skin, presenting with a flushed surface and lower firmness (**Figure 1A**). The infiltrating zone demonstrates greater sensitivity to treatment and yields superior therapeutic outcomes (2, 14, 17), and pathologically this zone represents the frontier of keloid tissue expansion and invasion towards the periphery (14, 18, 19). Building upon this, our study employed scRNA-seq on hypercellular and infiltrating zone tissue samples from four female patients (aged 18-60) with anterior chest keloids. The findings revealed that, beyond histological differences, the infiltration zone exhibits cellular heterogeneity characterized primarily by immune microenvironment dysregulation. Several specific genes and core regulatory pathways associated with the high invasiveness of the infiltration zone were identified. These results offer novel theoretical foundations for achieving precise, personalized treatment targeting distinct scar zones and for developing novel therapeutic targets (20).

## 2 Materials and methods

### 2.1 Patient recruitment and sample acquisition

Keloid samples were obtained from four patients who underwent keloid excision surgery at the Plastic Surgery Hospital of the Chinese Academy of Medical Sciences. The hypercellular and infiltrating zones were distinguished intraoperatively based on consistent clinical features: the hypercellular zone was identified as the elevated, dark red, and markedly firm region, whereas the infiltrating zone (margin) was defined as the flushed, less firm area at the junction between the keloid and normal skin. These criteria were derived from our previous clinical observations and published studies (12, 21, 22), ensuring reproducibility. The zonation was a literature-informed, predefined operational sampling framework designed specifically for this study. For each patient, paired specimens were collected from the infiltrating and hypercellular zones of the anterior chest keloid, yielding four specimens per zone and eight samples in total. None of the enrolled patients had received any topical or intralesional treatment (including corticosteroid injections, 5−fluorouracil, laser therapy, cryotherapy, or radiotherapy) targeting the keloid lesions prior to surgical excision (**Supplementary Table 1**). All experiments were approved by the Ethics Committee of the Plastic Surgery Hospital, (Chinese Academy of Medical Sciences and Peking Union Medical College, Beijing, China, approval number: 2024-86). All participating patients provided written informed consent. All ethical standards pertaining to human research participants were adhered to.

### 2.2 Sample pre-processing before sequencing

Keloid samples were preserved in GEXSCOPE tissue preservation solution and immediately transferred to the laboratory under cold conditions. Samples were washed three times with Hanks Balanced Salt Solution (HBSS), cut into 1-2mm fragments, and placed in 2ml GEXSCOPE tissue dissociation solution (Singleron Bio Com, Nanjing, China) for digestion. Following digestion, the mixture was filtered, centrifuged, and the supernatant discarded. The pellet was resuspended in 1 ml phosphate-buffered saline (PBS). After trypan blue (Sigma, United States) staining, cell viability and concentration (AO/PI) were recorded using a Countstar^®^ Rigel fluorescence cell counter.

### 2.3 Single-cell RNA sequencing

Single-cell suspensions (2×10^5^ cells/mL) with PBS were loaded onto microwell chip using the Singleron Matrix^®^ Single Cell Processing System. The scRNA-seq libraries were constructed according to the protocol of the GEXSCOPE^®^ Single Cell RNA Library Kits (Singleron) (23). Individual libraries were diluted to 4 nM, pooled, and sequenced on Illumina novaseq 6000 with 150 bp paired end reads. The raw reads were processed using fastQC and fastp to remove low-quality reads (24). The Poly-A tails and adapter sequences were removed using cutadapt. After quality control, the reads were mapped to the reference genome GRCh38 using STAR (25). The gene expression levels and UMI counts were statistically analyzed using the FeatureCounts function. An expression matrix file for subsequent analysis was generated based on the gene expression levels and UMI counts.

### 2.4 Quality control, dimensionality reduction and clustering

Cells were filtered using the following criteria: (i) genes expressed per cell < 200 or in the top 2% of gene counts were excluded; (ii) cells in the top 2% of UMI counts were removed; (iii) cells with mitochondrial transcript content > 30% were discarded; (iv) genes detected in fewer than 5 cells were excluded. After these filtering steps, 128,678 high−quality cells (65,947 from the infiltrating zone and 62,731 from the hypercellular zone) were retained for downstream analysis. We used functions from Seurat v3.1.2 for dimension-reduction and clustering (26). Then we used NormalizeData and ScaleData functions to normalize and scale all gene expression, and selected the top 2000 variable genes with FindVariableFeatures function for PCA analysis. Principal component analysis (PCA) was performed using the top 2,000 variable genes. The number of PCs to retain showed that the cumulative variance explained plateaued after 20 PCs. Adding more PCs introduced noise without improving cluster separation. Therefore, the first 20 PCs were used for subsequent clustering and Harmony integration. Batch effects between samples were corrected using Harmony v1.0 based on the top 20 principal components (PCs) from PCA. Harmony does not alter the raw gene expression matrix; it only adjusts the cell embedding coordinates. Successful removal of batch effects was confirmed by visualizing the integrated UMAP plots before and after correction (27). Clustering was performed using the Louvain algorithm with the Seurat function FindClusters. A resolution of 1.2 produced stable, biologically interpretable clusters that were reproducible across multiple runs and well−separated by canonical cell−type markers. Lower resolutions (≤1.0) resulted in over−aggregation of distinct cell types, while higher resolutions (≥1.5) yielded unstable subclusters without clear biological meaning. Unannotated cells were separated into 24 clusters by using Louvain algorithm and setting resolution parameter at 1.2. Cell clusters were visualized by using Uniform Manifold Approximation and Projection (UMAP) in a two-dimensional space. Reads were aligned to the human reference genome GRCh38 (Ensembl release 99), with gene annotation obtained from the Ensembl GTF file.

### 2.5 Differentially expressed gene analysis

To identify differentially expressed genes (DEGs), we used the Seurat FindMarkers function based on Wilcox likelihood-ratio test with default parameters, and selected the genes expressed in more than 10% of the cells in a cluster and with an average log (Fold Change) value greater than 0.25 as DEGs. To control for false positives due to multiple comparisons, adjusted p-values were calculated using the Bonferroni correction, and an adjusted p-value < 0.05 was applied as the statistical significance threshold for all DEG analyses. For the cell type annotation of each cluster, we combined the expression of canonical markers found in the DEGs with knowledge from literatures, and displayed the expression of markers of each cell type with heatmaps/dot plots/violin plots that were generated with Seurat DoHeatmap/DotPlot/Vlnplot function. Doublet cells were identified as expressing markers for different cell types, and removed manually.

### 2.6 Cell type annotation

The cell type identity of each cluster was determined with the expression of canonical markers found in the DEGs using SynEcoSys database. Heatmaps displaying the expression of markers used to identify each cell type were generated by Seurat v3.1.2 DoHeatmap.

### 2.7 Pathway enrichment analysis

To investigate the potential functions of DEGs, the Gene Ontology (GO) and Kyoto Encyclopedia of Genes and Genomes (KEGG) analysis were used with the “clusterProfiler” R package 3.16.1 (28). Pathways with p value less than 0.05 were considered as significantly enriched. Gene Ontology gene sets including molecular function (MF), biological process (BP), and cellular component (CC) categories were used as reference.

### 2.8 Trajectory analysis

Cell differentiation trajectory was reconstructed with Monocle2 and Monocle3 (29). Highly-variable genes (HVGs) were used to sort cells in order of spatial‐temporal differentiation. We used DDRTree to perform FindVariableFeatures and dimension-reduction. Finally, the trajectory was visualized by plot_cell_trajectory function. Next, CytoTRACE was used to predict the differentiation potential of monocyte subpopulations (30). Cell differentiation potential was assessed using CytoTRACE v0.3.3 (30), an algorithm designed to infer cellular differentiation states from single-cell RNA-seq data by leveraging the correlation between gene expression patterns and the total number of detected genes. Specifically, CytoTRACE first calculates the Gene Count Signature (GCS) by identifying the top 200 genes whose expression levels are most strongly correlated with the total number of genes expressed per cell. Subsequently, the algorithm employs non-negative least squares (NNLS) regression combined with a Markov diffusion process to smooth the GCS scores across the cell-cell similarity network, thereby robustly predicting the relative differentiation states and developmental trajectories of individual cells.

### 2.9 Cell-cell interaction analysis

The cell-cell interaction analysis was performed by CellPhoneDB v2.1.0 based on known receptor–ligand interactions between two cell types or subtypes (31). Cluster labels of all cells were randomly permuted for 1000 times to calculate the null distribution of average ligand-receptor expression levels of the interacting clusters. Individual ligand or receptor expression was thresholded with a cutoff value based on the average log gene expression distribution for all genes across all the cell types. The significant cell-cell interactions were defined as p value < 0.05 and average log expression > 0.1, which were visualized with the circlize v0.4.10 R package.

### 2.10 Histological analysis

Paraffin-embedded human keloid specimens were sectioned at 4 μm for histological evaluation. For general morphology, sections were stained with hematoxylin and eosin (H&E) following standard deparaffinization and rehydration. Collagen deposition was assessed on adjacent sections using Masson’s trichrome stain, which distinguishes collagen fibers (blue) from muscle and cytoplasm (red). After dehydration and mounting with neutral resin, all slides were examined and imaged under a light microscope.

### 2.11 Immunofluorescence

Following deparaffinization and antigen retrieval, tissue sections were treated with 3% hydrogen peroxide for 25 min at room temperature to inhibit endogenous peroxidase activity. After blocking with serum, the sections were incubated overnight at 4 °C in a humidified chamber with the following primary antibodies: anti-S100 (Servicebio, GB15359, 1:500), anti-IL−1β (Servicebio, GB11113, 1:500), anti-α−SMA (Servicebio, GB121364, 1:500), and anti-CD31 (Servicebio, GB153151, 1:500). Subsequently, the appropriate fluorophore-conjugated secondary antibodies were applied. Nuclei were counterstained with DAPI, and the sections were mounted with an anti-fade medium. Fluorescence images were captured using a confocal laser-scanning microscope (Carl Zeiss, Jena, Germany).

### 2.12 Immunohistochemical staining

Keloid tissue samples were promptly fixed in 4% paraformaldehyde. After routine processing involving graded dehydration and paraffin embedding, sections were cut and mounted. For immunohistochemistry, the sections were first deparaffinized and rehydrated, followed by antigen retrieval. Endogenous peroxidase activity was quenched by incubation with hydrogen peroxide. The sections were then incubated overnight at 4 °C with primary antibodies against CD68 (Servicebio, GB113150, diluted 1:500) and COL1 (Servicebio, GB115707, diluted 1:500). After washing, appropriate horseradish peroxidase (HRP)-conjugated secondary antibodies were applied. Color development was achieved using a 3,3’-diaminobenzidine (DAB) substrate. Finally, the sections were counterstained, dehydrated, cleared, and mounted with neutral resin for microscopic examination and image acquisition.

## 3 Results

### 3.1 Overall cellular diversity and heterogeneity of keloid infiltrating and hypercellular zones by scRNA-seq

We performed scRNA-seq on a total of eight samples of the hypercellular zones and infiltrating zones of keloids resected from four patients, and conducted a comparative analysis (**Figure 1A**, **Supplementary Table 2**). After quality control, a total of 128,678 single-cell transcriptomes were profiled, comprising 65,947 cells from the infiltrating zone and 62,731 cells from the hypercellular zone. Dimensionality reduction and clustering partitioned these cells into 23 clusters (**Figure 1B**) (32). In cell annotation, based on established cell marker genes (18, 33, 34), these cells were assigned to ten cell clusters: fibroblasts, keratinocytes, endothelial cells, mononuclear-phagocytes, Schwann cells, mast cells, mural cells, T and NK cells, melanocytes, and B cells (**Figure 1B**, **Supplementary Table 3**). Among these, fibroblasts constituted the largest proportion (74.15%), followed by endothelial cells (7.15%) and keratinocytes (5.21%). The heatmap shows the dominant marker genes of each cell type (**Figure 1C**, **Supplementary Figure 1A**) revealed the transcriptional heterogeneity across different cell clusters. Additionally, we projected the distribution of marker genes onto the UMAP plot (**Figure 1D**). Subsequent analysis of cell type proportions across samples and between hypercellular and infiltrating zones revealed marked compositional differences (**Figures 1F, G**). The infiltrating zones were enriched in mononuclear-phagocytes and endothelial cells, whereas the hypercellular zone showed fibroblast predominance (**Figures 1F, G**). These differences suggest that the observed disparities in invasiveness and treatment sensitivity between the infiltrating and hypercellular zone may arise from cellular heterogeneity, particularly the abundance and distribution of fibroblasts, mononuclear-phagocytes, and endothelial cells.

### 3.2 Fibrotic features and fibroblasts heterogeneity of keloid infiltrating and hypercellular zones

**Figure 2:**
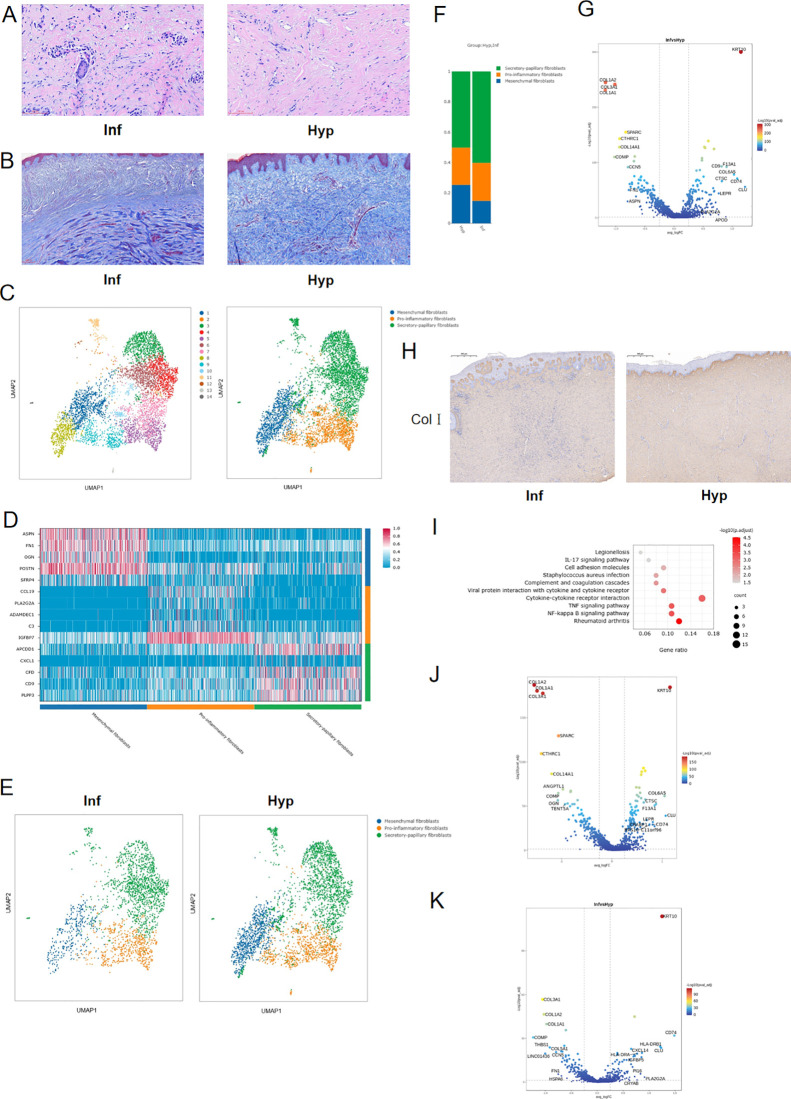
Differences in fibrosis severity and fibroblasts subpopulation composition between the two zones. **(A)** Histological H&E staining of the two zones of human keloid. Scale bar: 100 μm. **(B)** Histological Masson staining of the two zones. Scale bar: 200 μm. **(C)** The UMAP plot of fibroblasts unbiased clustering results and fibroblasts subpopulation clustering results. **(D)** Heatmap of DEGs of fibroblasts subpopulations. **(E)** The distribution of fibroblasts subpopulation derived from two groups samples in UMAP plot. **(F)** Proportion plot of fibroblasts subpopulation composition between two groups and eight samples. **(G)** Volcano plot of DEGs in fibroblasts between the two zones. **(H)** Immunohistochemical staining of collagen I in the two zones. Scale bar: 500 μm. **(I)** KEGG pathway enrichment analysis plot of fibroblasts. **(J)** Volcano plot of DEGs in SPFs between the two zones. **(K)**Volcano plot of DEGs in MFs between the two zones.

To investigate whether fibrotic and fibroblast heterogeneity underlie the zonal disparities in keloid invasiveness and treatment response (35, 36), we performed H&E and Masson staining on tissues from the two zones. Compared with the hypercellular zone, the infiltrating zone exhibited lower fibroblast density, more inflammatory cells and newly formed capillaries (**Figure 2A**), consistent with single-cell sequencing results showing distinct distributions of fibroblasts, monocyte-macrophages, and endothelial cells (**Figures 1F, G**). Masson staining revealed densely packed, regularly arranged collagen fibers in the hypercellular zone, whereas collagen fibers were looser and irregular in the infiltrating zone (**Figure 2B**).

Unsupervised clustering of fibroblasts identified 14 distinct clusters (**Figure 2C**). Based on previous studies (15, 17, 37), these clusters were annotated into three subtypes: secretory-papillary fibroblasts (SPFs), mesenchymal fibroblasts (MFs), and pro-inflammatory fibroblasts (PFs) (**Figure 2C**). Secretory-reticular fibroblasts (SRFs) were not classified separately due to extremely low cell counts. The heatmap showed the major marker genes of the three fibroblast subtypes (**Figure 2D**). The proportions of the three fibroblast subtypes were markedly different between the infiltrating and hypercellular zones, with a significantly higher proportion of SPFs and a lower proportion of MFs in the infiltrating zone (**Figures 2E, F**). DEGs revealed an immunomodulatory signature in infiltrating zone fibroblasts, with upregulation of CD74 and CTSC, resembling inflammatory cancer-associated fibroblast (iCAF) (18, 38, 39). In contrast, hypercellular zone fibroblasts upregulated collagen-related genes (COL1A1, COL1A2, COL3A1) and ECM associated genes (SPARC, CTHRC1, COMP) (40) (**Figure 2G**), consistent with COL1 staining (**Figure 2H**). KEGG pathway analysis further revealed that DEGs in infiltrating zone fibroblasts were significantly enriched in TNF signaling, NF-κB signaling, and cytokine-cytokine receptor interaction pathways, indicating an active inflammatory and immune communication state in infiltrating zone fibroblasts (41) (**Figure 2I**). These findings suggest that infiltrating zone fibroblasts are associated with immune dysregulation and may contribute to invasion, whereas hypercellular zone fibroblasts show gene expression patterns consistent with ECM production and deposition. To further delineate the cellular heterogeneity underlying these zone-specific functional patterns, we further examined the two fibroblast subtypes that exhibited the most pronounced differential expression: SPFs and PFs. In SPFs, the hypercellular zone showed significant upregulation of classical ECM components and matrix-remodeling genes, including COL1A1, COL1A2, COL3A1, SPARC, CTHRC1, and COMP (**Figure 2J**) (15, 42), whereas the infiltrating zone did not show enrichment of immunomodulatory genes. This expression pattern suggests that SPFs may represent a major cellular source of the ECM-producing phenotype observed in the hypercellular core, consistent with previous reports identifying SPFs as a distinct keloid fibroblast subpopulation with matrix-producing capacity (15, 42). In contrast, PFs in the infiltrating zone exhibited a distinctive immunomodulatory signature, with upregulation of HLA-DRB1, CD74, and CXCL14 (**Figure 2K**). HLA-DRB1 and CD74 are known to be involved in antigen presentation, and CXCL14 has been implicated in monocyte recruitment and M2-like macrophage polarization (39, 43). The enrichment of these genes in PFs is consistent with the possibility that PFs contribute to the immunomodulatory, iCAF-like phenotype in the keloid margin. In contrast, MFs did not show pronounced zone-specific differential gene expression (**Supplementary Figure 1C**).

In summary, fibroblasts exhibit region-specific phenotypes: immune regulation and invasiveness in the infiltrating zone versus matrix synthesis, collagen deposition, and core sclerosis in the hypercellular zone, contributing to the distinct pathological features of each zone. SPFs are primarily associated with ECM accumulation in the hypercellular zone, whereas PFs may play a role in immune crosstalk and the inflammatory microenvironment of the infiltrating zone.

### 3.3 Mononuclear phagocytes transcriptomes reveal distinct immune landscapes of infiltrating and hypercellular zones

**Figure 3:**
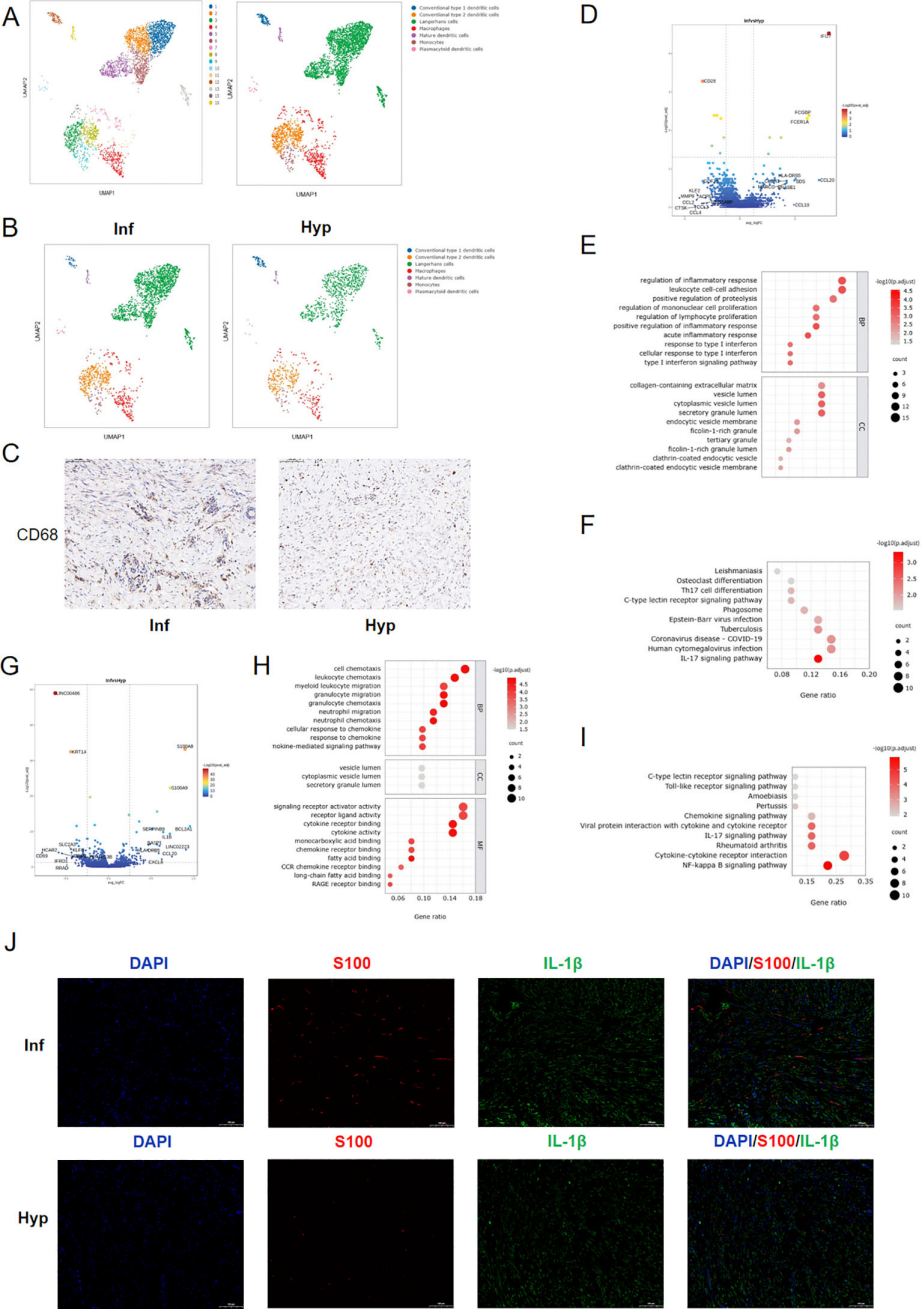
Transcriptional profiles of mononuclear phagocytes revealed the immune characteristics of infiltrating and hypercellular zones. **(A)** The UMAP plot of mononuclear phagocytes unbiased clustering results and mononuclear phagocytes subpopulation clustering results based on marker genes. **(B)** The distribution of mononuclear phagocytes subpopulation derived from two groups samples. **(C)** Immunohistochemical of CD68 in the two zones. Scale bar: 100 μm. **(D)** Volcano plot of DEGs in macrophages between the two zones. **(E)** GO pathway enrichment analysis plot of macrophages. **(F)**KEGG pathway enrichment analysis plot of macrophages. **(G)** Volcano plot of DEGs in LCs between the two zones. **(H)** GO pathway enrichment analysis plot of LCs. **(I)** KEGG pathway enrichment analysis plot of LCs. **(J)** Immunofluorescence staining of S100(red) and IL-1β(green) in the dermis of infiltrating and hypercellular zones in keloids. Scale bar: 100 μm.

Given the critical role of the immune microenvironment in shaping zone-specific pathological features and treatment responses (17, 40), we characterized the transcriptional landscapes of mononuclear phagocytes, which revealed distinct immune signatures between the infiltrating and hypercellular zones. Through unsupervised clustering and cell annotation, the 16 clusters obtained from mononuclear phagocytes were classified into seven cell types (17, 44, 45): Langerhans cells (LCs), macrophages, conventional type 1 dendritic cells, conventional type 2 dendritic cells, mature dendritic cells, plasmacytoid dendritic cells, and monocytes (**Figure 3A**). The heatmap showing the major marker genes of distinct immune cell types (**Supplementary Figure 2A**). Immune infiltration differed markedly between the two zones, particularly with enrichment of macrophages and Langerhans cells in the infiltrating zone (**Figure 3B**; **Supplementary Figures 2B, C**). Immunohistochemical staining for CD68 further confirmed the differences in immune infiltration between the two zones (**Figure 3C**).

In DEGs analysis of macrophages, the concurrent upregulation of FCER1A and FCGBP suggests that immunoglobulin-mediated immune activation may represent a previously underappreciated mechanism in keloid pathogenesis (**Figure 3D**), potentially linking atopic predisposition to lesional inflammation. More importantly, IFI27 was significantly upregulated in the infiltrating zone (**Figure 3D**), and GO analysis revealed significant enrichment of pathways related to type I interferon signaling (**Figure 3E**), which aligns with recent evidence implicating sustained interferon responses in pathological fibrosis, including liver fibrosis (46), vascular fibrosis (47), and scleroderma (48). Additionally, pathways related to inflammatory response regulation and lymphocyte proliferation regulation were also enriched (**Figure 3E**), indicating an active immune microenvironment in the infiltrating zone. KEGG analysis further identified IL-17 signaling pathway as the most prominently enriched pathways (**Figure 3F**). Consistent with previous studies demonstrating the pro-fibrotic role of Th17 cells in keloids (44, 49), the enrichment of IL-17 signaling in infiltrating zone macrophages suggests their potential involvement in amplifying inflammatory responses and promoting fibroblast activation in the keloid margin. Collectively, these findings delineate a functional landscape in which infiltrating zone macrophages exhibit an interferon-activated and antibody-associated phenotype. This transcriptional signature suggests that infiltrating zone macrophages contribute to a chronic inflammatory microenvironment reminiscent of certain infectious and autoimmune-like conditions, which may drive the invasive growth characteristic of keloid margins (50).

**Figure 4:**
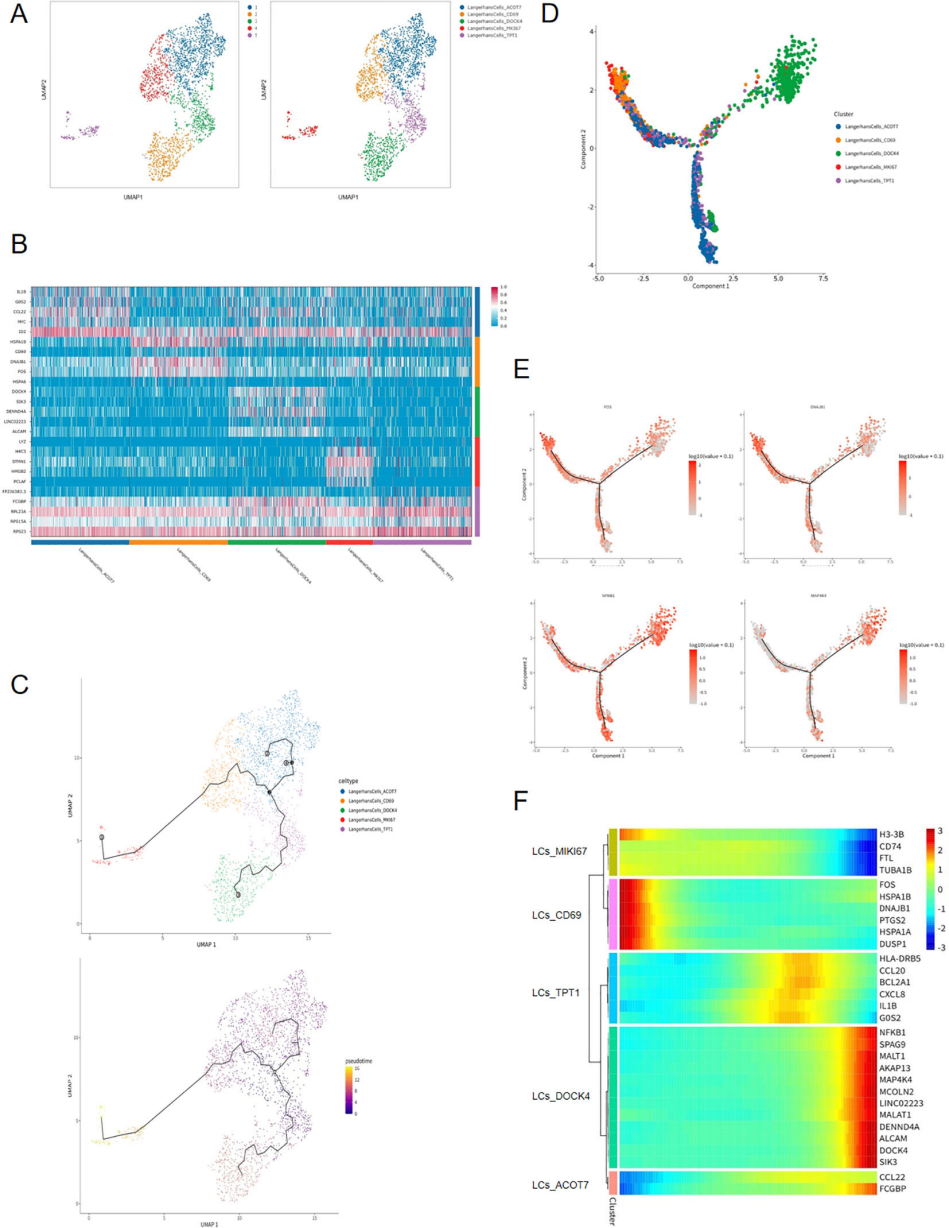
Clustering and transcriptional features of LCs. **(A)** The UMAP plot of LCs unbiased clustering results and subpopulation clustering results. **(B)** Heatmap of DEGs in LCs_subpopulations. **(C)** Pseudotime analysis of the sequential relationship of LCs subgroups in development. Dark blue is the beginning of differentiation and yellow is the end point. **(D)** Pseudo-time ordering of LCs subgroups revealed branching trajectories. **(E)** Major DEGs of LCs subgroups displayed at the trajectories plot. **(F)** Hierarchical clustering of branching-dependent genes revealed 5 gene modules and displayed the expression of representative genes for each gene module over pseudo-time.

Functional annotation of infiltrating zone LCs revealed a coordinated program centered on chemokine-mediated immune cell recruitment. The most significantly enriched biological processes included neutrophil and granulocyte chemotaxis, myeloid leukocyte migration, and chemokine-mediated signaling (**Figure 3H**). These findings align with the upregulation of inflammatory cytokines such as S100A8/A9, IL-1β and CCL20 (**Figure 3G**), which collectively construct an active regulatory network in the immune microenvironment (44, 51, 52) of infiltrating zone, and immunofluorescence staining further confirmed the high expression of S100 proteins and IL-1β in the infiltrating zone (**Figure 3K**). The concurrent enrichment of IL-17 signaling and NF-κB pathways supports that LCs recruit Th17 cells via CCL20 and subsequently amplify local inflammation through NF-κB-dependent cytokine production (**Figure 3I**). Collectively, these results position infiltrating zone LCs as sentinels and amplifiers of the keloid margin immune microenvironment: they orchestrate neutrophil and Th17 cell infiltration, and sustain local inflammation through a self-amplifying cytokine/chemokine network. LCs exhibit marked heterogeneity in distribution and function between the infiltrating and hypercellular zones, and may serve as a key cell type associated with the heightened immune response in the infiltrating zone. Further subclustering of LCs identified five distinct subpopulations (**Figure 4A**, **Supplementary Figure 2D**). The proportions of these five subpopulations showed no significant differences between the infiltrating and hypercellular zones (**Supplementary Figure 2E**). The heatmap displays the major DEGs across the five subpopulations (**Figure 4B**). To investigate the pseudotemporal ordering trajectory of LCs within the keloid immune microenvironment, we performed pseudotime analysis. The results showed that LCs_CD69 localized to the pseudotime-early position, whereas LCs_DOCK4 resided at the pseudotime-late position (**Figures 4C, D**). LCs_ACOT7 was present throughout the pseudotime continuum and constituted a relatively high proportion of cells, while LCs_TPT1 occupied a pseudotime-intermediate position (**Figures 4C, D**). Further analysis revealed a three pseudotime-ordered states of LCs in keloid tissue. The pseudotime-early state LCs_CD69 highly expressed stress-responsive genes (HSPA1A, DNAJB1, FOS, DUSP1) (**Figures 4E, F**), indicating an activated state following tissue insult (53, 54). The pseudotime-intermediate state LCs_TPT1 upregulated antigen presentation-associated genes (HLA-DRB5), chemokines (CCL20, CXCL8), and pro-inflammatory cytokines (IL1B) (**Figure 4F**), suggesting a role in immune cell recruitment and inflammation amplification (55). The pseudotime-late state LCs_DOCK4 enriched genes involved in NF-κB signaling (NFKB1, MALT1) and cell migration (DOCK4, ALCAM) (**Figures 4E, F**), representing a gene expression pattern consistent with a potential effector function (56, 57). These findings delineate a pseudotemporal continuum from stress-responsive to immune-related gene expression patterns. Integrating the global transcriptomic profiles of LCs, the pseudotime-late subpopulation LCs_DOCK4, characterized by high NFKB1 expression, may play a pivotal effector role in orchestrating the local inflammatory network in keloids, suggesting NF-κB signaling as a potential research direction and therapeutic target.

### 3.4 Cell communication features and transcriptional differences of other cells

**Figure 5:**
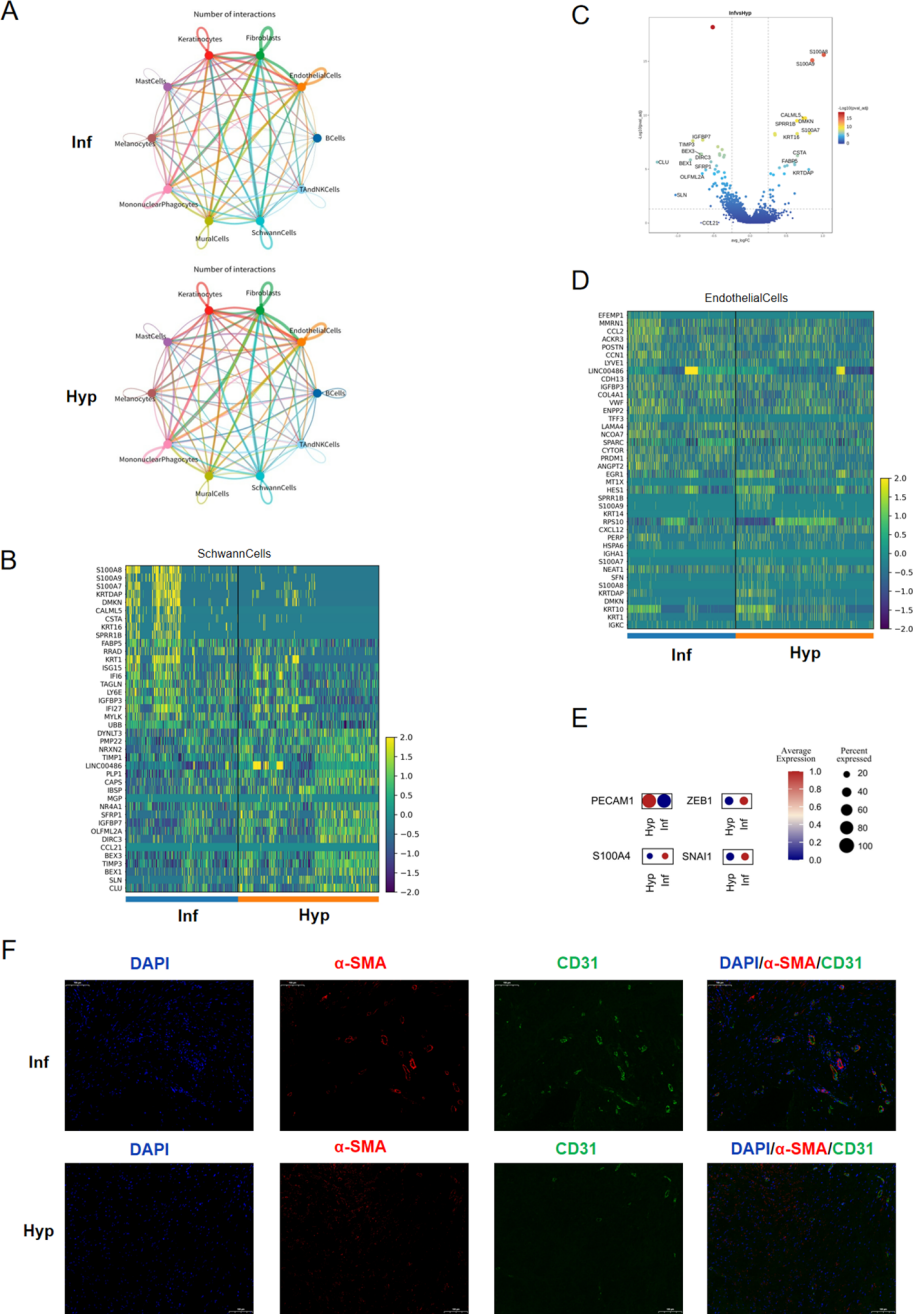
Intercellular communication and transcriptomic features of endothelial and Schwann cells. **(A)** Interaction network of major cell types in the two zones. Edge thickness indicates the total number of ligand-receptor pairs, and line color corresponds to the ligand-expressing cell type. **(B)** Heatmap of DEGs in endothelial cells between the two zones. **(C)** Volcano plot of DEGs in Schwann cells between the two zones. **(D)** Heatmap of DEGs in Schwann cells between the two zones. **(E)** Bubble plot comparing the expression of EndMT-related genes in endothelial cells. Bubble color represents expression level (blue: low, red: high), and bubble size indicates the percentage of cells expressing the gene. **(F)** Immunofluorescence staining of α-SMA(red) and CD31(green) in the dermis of infiltrating and hypercellular zones in keloids. Scale bar: 100 μm.

We employed CellPhoneDB to investigate intercellular communication among major cell types in the infiltrating and hypercellular zones of keloid tissues, in which dense intercellular communication networks were observed among and within fibroblasts, Schwann cells, and endothelial cells in both infiltrating and hypercellular zones (**Figure 5A**; **Supplementary Figures 3A–C**). We further examined zone-resolved ligand-receptor pairs centered on fibroblasts, endothelial cells, and Schwann cells (**Supplementary Figures 4A–C**). In the infiltrating zone, fibroblast-centered interactions were associated with inflammatory and chemotactic axes, including CXCL1-ACKR1, RARRES2-CCRL2, ANXA1-FPR1, CD99-PILRA, and THY1-ADGRE2 (**Supplementary Figure 4A**) (58, 59). In contrast, hypercellular-zone fibroblast-centered interactions included growth-factor and matrix-remodeling axes such as WNT5A-ROR1/2, PDGFD-PDGFRB/PDGFR complex, and FGF7-FGFR2 (60, 61). Endothelial-cell-centered interactions in the infiltrating zone included CXCL8-ACKR1, CCL14/CCL23-CCR1, VEGFC-KDR, and PGF-FLT1 complex, consistent with a potential role in inflammatory vascular remodeling and EndMT-associated communication (**Supplementary Figure 4B**) (35, 59). Hypercellular-zone endothelial cells retained vascular and developmental signaling axes, including PGF-FLT1, ADM-RAMP3, and APP-PLXNA4/TNFRSF21 (62, 63). Schwann cells also exhibited zone-resolved communication differences (**Supplementary Figure 4C**). In the infiltrating zone, Schwann-cell-centered interactions featured NRXN1/2-DAG1, TENM3-ADGRL2, L1CAM-Ezrin and CD99-PILRA, consistent with a potential more plastic neuro-immune and matrix-interactive communication state (64, 65). In contrast, the hypercellular zone showed SLITRK6-PTPRS, PTHLH-PTH2R and APP-PLXNA4 interactions, consistent with a growth-factor and tissue-remodeling-oriented communication program, although functional validation is required (66–68).

Next, we analyzed the transcriptional profiles of Schwann cells and endothelial cells in the two zones. DEGs analysis of Schwann cells revealed distinct functional states between the two keloid zones. Schwann cells in the infiltrating zone were markedly activated, as evidenced by upregulation of S100A, while downregulation of TIMP3 (**Figures 5C, D**) relieved the inhibition of matrix metalloproteinases (MMPs), thereby ECM degradation (69). Moreover, upregulation of genes such as KRT16, SPRR1B and CSTA (**Figures 5C, D**) suggested a phenotypic shift toward an epithelial-like and fibroblast-like state (70, 71). These findings are consistent with the possibility that Schwann cells at the keloid margin undergo activation and phenotypic plasticity, potentially contributing to invasive growth through enhanced matrix remodeling and intercellular interactions, consistent with previous studies implicating Schwann cells in promoting fibrosis in keloids (70, 71). What’s more, recent studies have demonstrated that endothelial to mesenchymal transition (EndMT) is markedly present in keloid tissues and represents a critical factor contributing to the high invasiveness of keloids (19, 35). In our study, infiltrating zone endothelial cells exhibited an EndMT-associated transcriptional signature, characterized by upregulation of pro-EndMT genes (ZEB1, S100A4, SNAI1, POSTN) and downregulation of the endothelial marker PECAM1(**Figures 5E, F**) (72, 73). Immunofluorescence results further confirmed evident colocalization of CD31 and α-SMA in the infiltrating zone (**Figure 5G**). This provides critical transcriptomic evidence for the spatial heterogeneity of EndMT in keloids. It not only corroborates the active EndMT process in the infiltrating zone at the molecular level, but also provides a preliminary foundation for considering EndMT as a potential mechanism that may drive invasive growth for the invasive growth of keloids.

## 4 Discussion

Keloids represent a formidable clinical challenge due to their aggressive, tumor-like growth and high recurrence rates (74). Previous transcriptomic studies have largely treated keloids as homogenous fibrotic masses, obscuring the intricate spatial dynamics that drive lesion expansion (75). By meticulously dissecting the keloid tissue into the hypercellular and infiltrating zones, our scRNA-seq analysis uncovers a profound spatial and functional dichotomy. This spatially resolved single cell atlas reveals that the invasive margin of keloids functions as an immunology driven niche, which is a highly active, immune-orchestrated microenvironment, not merely a passive front of collagen deposition, fundamentally distinct from the fibrotic core (76). Within the limitations of this study, our findings suggest a shift of the paradigm of keloid pathogenesis from a purely fibroblast-centric model to an immunology-dominated integrated landscape in which immune dysregulation and cellular plasticity actively drive disease progression (41). We strikingly observed that the compositional dichotomy between the two zones. The hypercellular zone is dominated by ECM producing myofibroblasts, whereas the infiltrating zone is enriched in mononuclear phagocytes and endothelial cells. This immune rich architecture may contribute to the clinically observed higher treatment sensitivity of the infiltrating zone, as immune enriched microenvironments are often more pharmacologically accessible (77–79). Histologically, the loose, irregular collagen network in the infiltrating zone provides a permissive structure for cellular invasion, in contrast to the dense, organized matrix of the core that may impede drug penetration (14, 80, 81).

A central advance of our study is the identification of distinct spatial phenotypes within the fibroblast population. While hypercellular zone fibroblasts exhibit classic myofibroblast features—characterized by profound ECM synthesis and collagen deposition (COL1A1, SPARC)—fibroblasts in the infiltrating zone adopt an immunomodulatory phenotype (18, 82). The upregulation of CD74, CTSC and CD9 in the infiltrating zone closely parallels the signature of iCAFs observed in the tumor microenvironment (18, 38, 39, 83). This raises the possibility that at that at the invasive margin, fibroblasts may primarily function to recruit and modulate immune cells rather than solely producing matrix.

The hyper-inflamed state of the infiltrating zone is driven by a highly coordinated mononuclear phagocyte network. Our discovery of a prominent Type I interferon (IFN) signature (IFI27) alongside Ig-mediated activation (FCER1A) in infiltrating zone macrophages reveals a chronic inflammatory state with features reminiscent of certain autoimmune conditions (84, 85), adding single-cell transcriptomic evidence to previous reports of immune dysregulation and autoimmunity-like features in keloids (86, 87). Crucially, we mapped a pseudotime-ordered sequence of LCs specific to the keloid margin. Ordered along pseudotime from a stress-responsive state (CD69^+^) to a terminal, NF-κB-driven effector state (DOCK4^+^), these LCs show gene expression patterns consistent with orchestrating the recruitment of neutrophils and Th17 cells via a robust chemokine network (CXCL8, CCL20, IL-1β) (86–88). It should be noted that pseudotime analysis from cross-sectional data infers a possible ordering of cells but cannot definitively establish true developmental progression, and alternative trajectory orders cannot be ruled out. Therefore, the pseudotime-ordered states described here are correlative and hypothesis-generating. The significant enrichment of the IL-17 signaling pathway within this niche suggests the possibility of a self-amplifying feedback loop where immune cells and iCAF-like fibroblasts sustain a chronic inflammatory frontier (38, 39, 89), potentially driving continuous peripheral invasion (44, 90, 91).

Beyond fibroblasts and immune cells, our data elucidate profound phenotypic plasticity in supporting stromal cells at the keloid margin. We provide definitive transcriptomic and spatial evidence of EndMT in the infiltrating zone, marked by the upregulation of ZEB1, S100A4, SNAI1 and downregulation of PECAM1, and confirmed by CD31/α-SMA colocalisation (19, 35). This mesenchymal activation likely contributes to vascular remodeling and generation of fibroblast like cells that could augment matrix production. Concurrently, Schwann cells in this region demonstrate marked activation (S100B) and a shift toward a fibroblast-like state, facilitating ECM remodeling via the downregulation of TIMP3, consistent with recent reports of repair-like, pro-fibrotic Schwann cells in keloids (69–71). These multi-lineage transitions mirror the aggressive tissue-remodeling processes seen in malignant invasion, solidifying the concept that keloid peripheral growth is a multi-cellular, microenvironment-driven event.

Collectively, the infiltrating zone operates as a tumor-like, highly communicative ecosystem in which macrophages, Langerhans cells, EndMT derived cells, and plastic Schwann cells cooperate to sustain chronic inflammation. This integrated view has direct clinical implications. Targeting immune components—particularly the IL-17, type I interferon, and NF-κB pathways—may be more effective at the invasive margin than suppressing fibroblasts alone (92–94). Moreover, zone-specific markers such as CD9, VCAN, and NFKB1 could serve as biomarkers for treatment response and disease monitoring (50, 95).

Despite these novel insights, our study has several limitations that warrant consideration. First, our study included only four patients, all female with anterior chest keloids. This constitutes a major limitation that constrains the generalizability of our findings. Keloid pathogenesis, immune microenvironment composition, and treatment responses are known to vary substantially by anatomical site, sex, genetic background, and disease duration. Therefore, the cellular and molecular landscapes described here may not be fully representative of keloids at other locations (e.g., earlobe, shoulder), male patients, or diverse ethnic populations. Future studies with larger, multi−site, sex−balanced, and ancestrally diverse cohorts are essential to validate the universality of the observed zone−specific immune−stromal signatures. Second, while our macroscopic dissection of the hypercellular and infiltrating zones effectively captured regional dichotomies, it lacks the continuous, high-resolution architectural context that cutting-edge spatial transcriptomics could provide. And the innermost aging zone, which is hypocellular and densely sclerotic, was not analyzed in this study because it is biologically quiescent and less relevant to active invasion and treatment response. Future spatially resolved studies are warranted to characterize this region separately. Finally, the present study is primarily descriptive, relying on transcriptomic profiling and histological confirmation. All inferred relationships between specific cell subpopulations, gene expression signatures, and keloid pathogenesis are correlative and hypothesis−generating. Functional validation through *in vitro* or *in vivo* experiments is required before any causal conclusions can be drawn. Addressing these gaps will be a pivotal next step toward translating these transcriptomic discoveries into clinical precision therapies. Otherwise, we acknowledge that paired pre-and post-treatment biopsies were not obtained due to ethical concerns regarding wound healing impairment caused by 5-fluorouracil. Future prospective studies with optimized regimens or sampling strategies are needed to directly validate the causal relationship between the identified zone-specific features and treatment response.

In conclusion, in this cohort of four female patients with anterior chest keloids, this study redefines the keloid margin as an immunology-dominated, highly interactive niche. By delineating zone-specific cellular states and molecular programs, we provide a preliminary theoretical foundation for considering precision immunotherapies aimed at halting keloid expansion at its invasive front. Larger and more diverse cohorts are required to validate the generalizability of these findings.

## Data Availability

The datasets presented in this study have been deposited in the NCBI Gene Expression Omnibus (GEO) repository under accession number GSE335482.
