# Supplementary figures and images for: Immune-dominated cellular heterogeneity and stromal plasticity in keloid infiltrating and hypercellular zones revealed by single-cell RNA sequencing
Source: Front Immunol. 2026 Jun 26;17:1873878. doi: 10.3389/fimmu.2026.1873878 (PMC13349762; doi:10.3389/fimmu.2026.1873878)

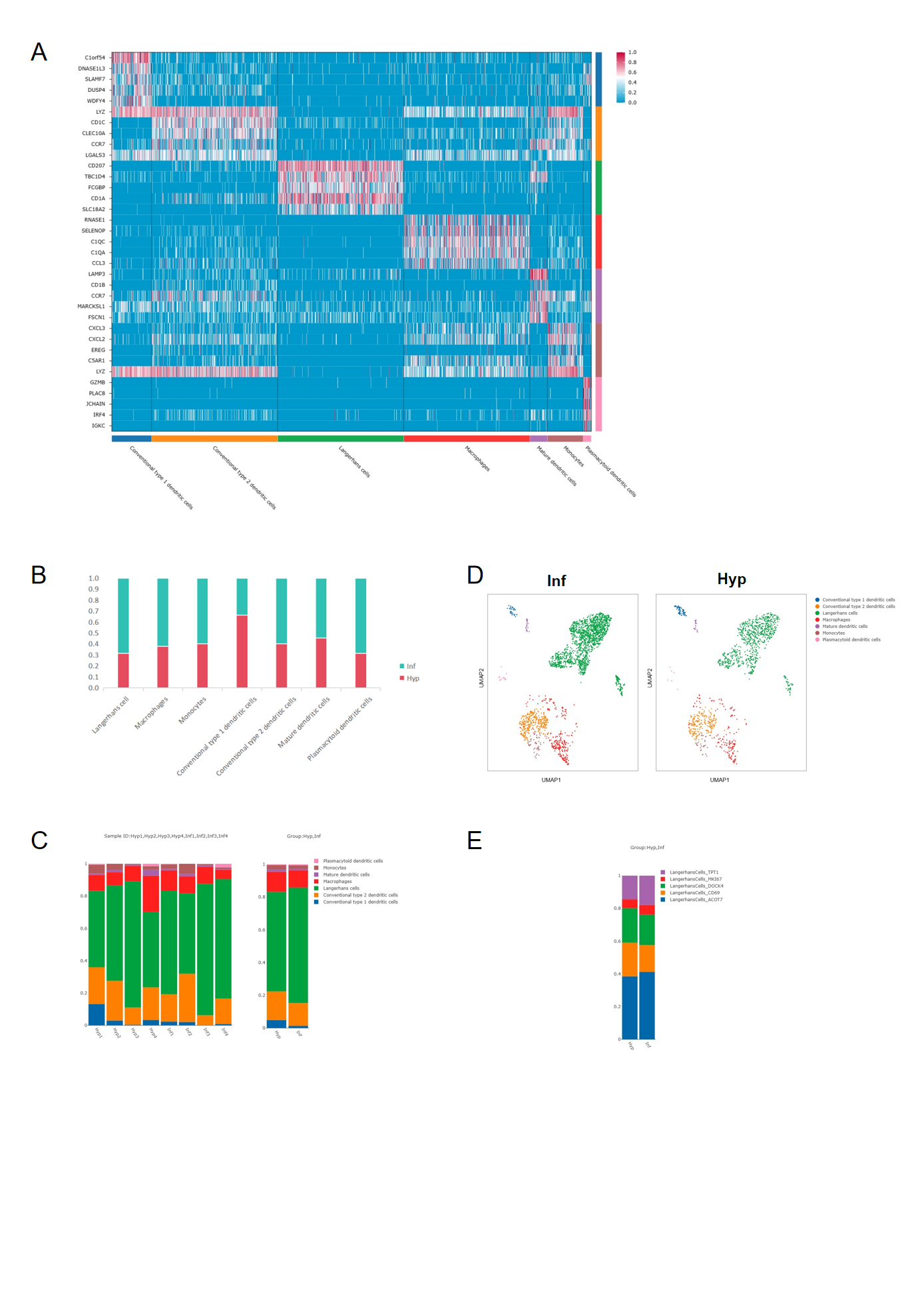

Supplement: Supplementary file 1 [file Image1.tif]

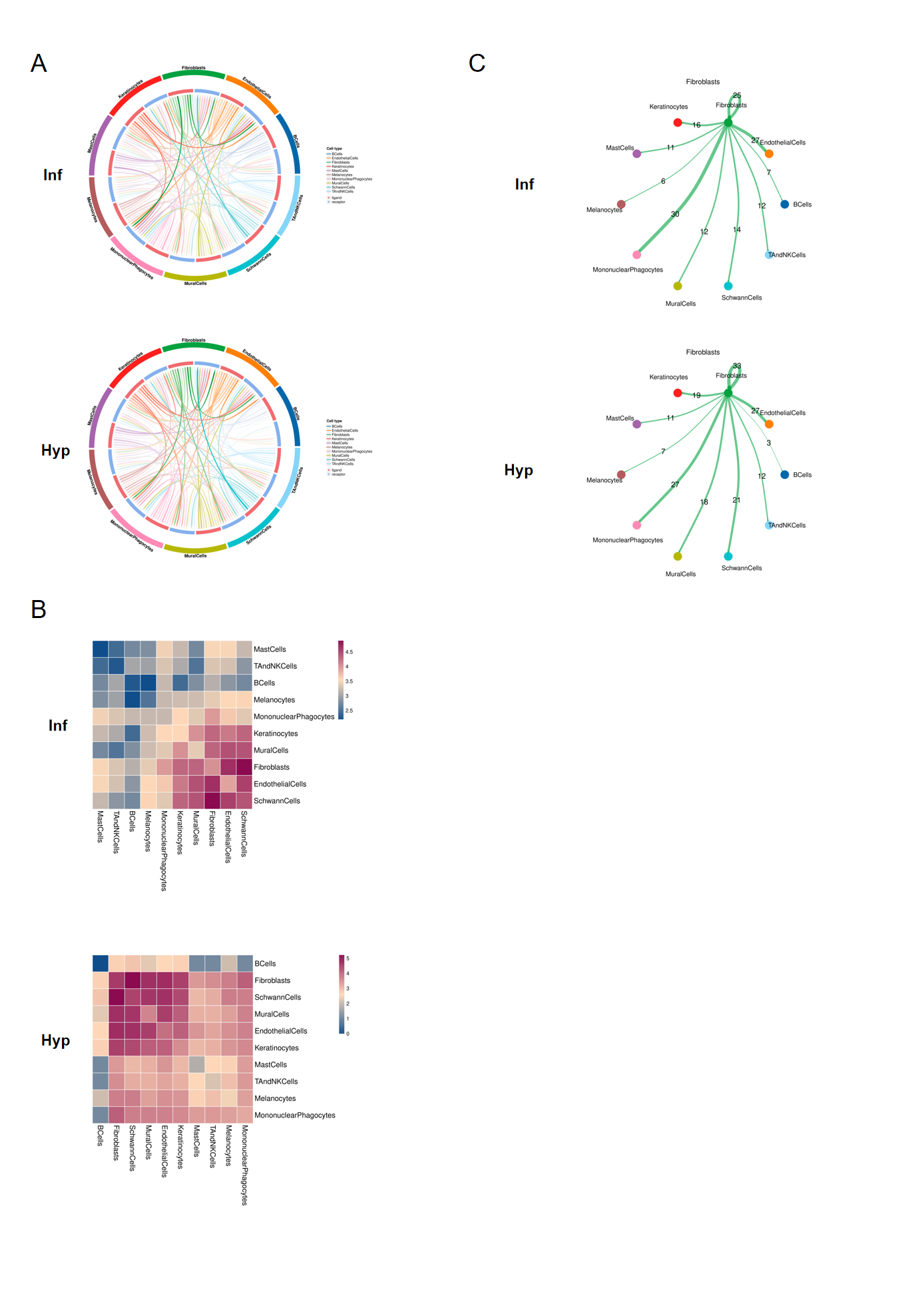

Supplement: Supplementary file 2 [file Image2.tif]

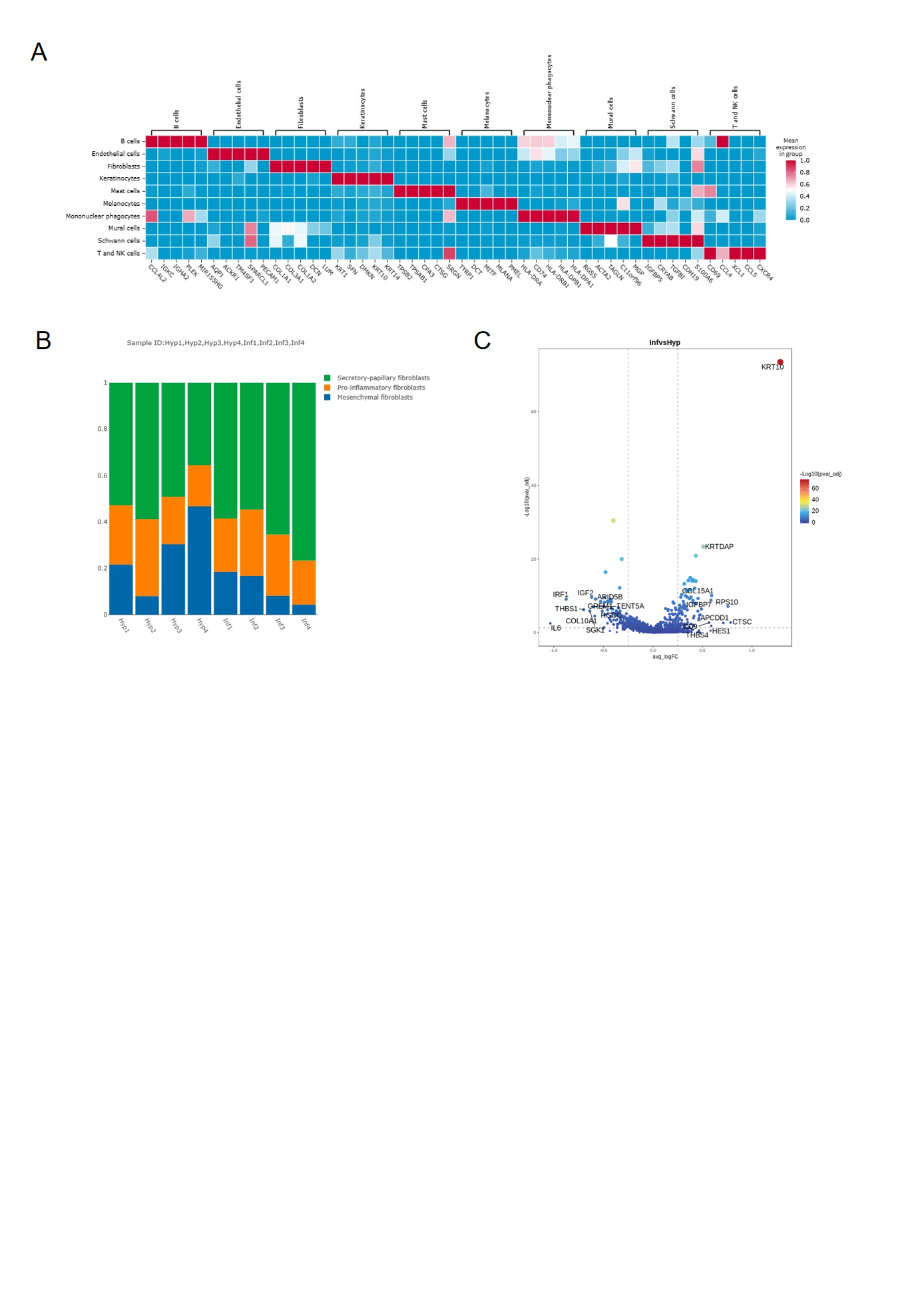

Supplement: Supplementary file 3 [file Image3.tif]

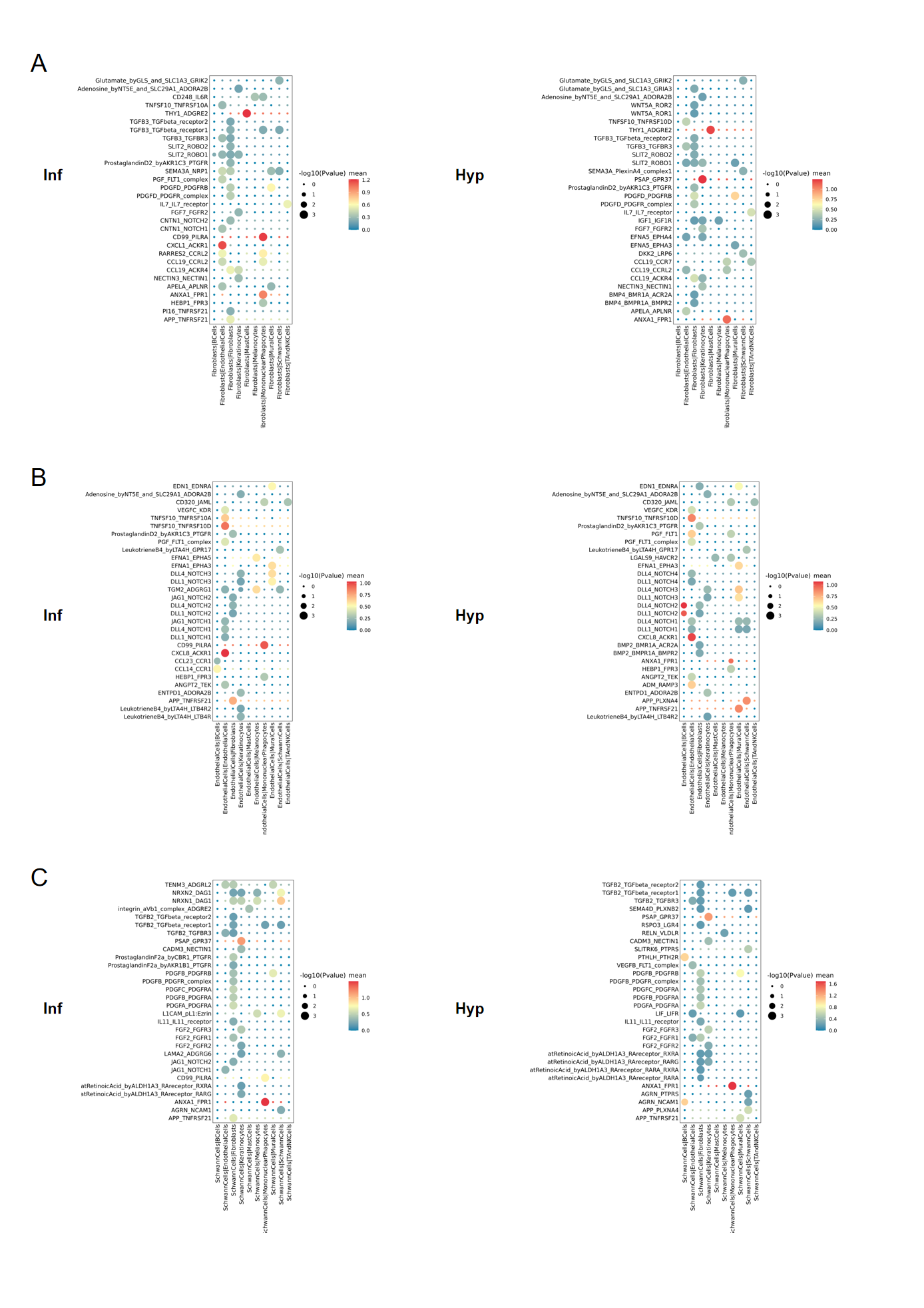

Supplement: Supplementary file 4 [file Image4.tif]
