# Supplementary material for: Immune-dominated cellular heterogeneity and stromal plasticity in keloid infiltrating and hypercellular zones revealed by single-cell RNA sequencing
Source: Front Immunol. 2026 Jun 26;17:1873878. doi: 10.3389/fimmu.2026.1873878 (PMC13349762; doi:10.3389/fimmu.2026.1873878)
Supplement: Supplementary file 5 [file SupplementaryFile1.docx]

Supplementary Figure.1 (A)Heatmap of top 5 DEGs of all major cell types. (B) Proportion plot of fibroblasts subpopulation composition between eight samples. (C)Volcano plot of DEGs in MFs between the two zones.

Supplementary Figure.2 (A)Cluster heatmap of DEGs of mononuclear phagocytes subpopulation. (B)The distribution of mononuclear phagocytes subpopulations derived from two groups samples. (C)Proportion plot of mononuclear phagocytes subpopulation composition between two groups and eight samples. (D)The distribution of mononuclear phagocytes subpopulation derived from two groups samples in UMAP plot. (E)Proportion plot of LCs subpopulation composition between two groups.

Supplementary Figure.3 (A)Chord diagram of cells i**nteraction in the infiltrating and hypercellular zones. (B)Heatmap showing the numbers of interpopulation communications with each other in the two zones. (C)Cellular communication network diagram between fibroblasts** and other cell types. **Different colors represent cell types, and the numbers indicate the number of ligand-receptor pairs.**

Supplementary Figure.4 (A)Zone-resolved CellPhoneDB ligand-receptor interactions centered on fibroblasts. (B)Zone-resolved CellPhoneDB ligand-receptor interactions centered on endothelial cells. (C)Zone-resolved CellPhoneDB ligand-receptor interactions centered on Schwann cells. Dot size represents the mean −log^10^(P value), and color indicates the corresponding interaction significance/intensity metric displayed in each panel. Because CellPhoneDB infers ligand-receptor co-expression rather than direct functional causality, these interactions were interpreted as candidate communication axes.
