# Supplementary material for: Immune-dominated cellular heterogeneity and stromal plasticity in keloid infiltrating and hypercellular zones revealed by single-cell RNA sequencing
Source: Front Immunol. 2026 Jun 26;17:1873878. doi: 10.3389/fimmu.2026.1873878 (PMC13349762; doi:10.3389/fimmu.2026.1873878)
Supplement: Supplementary file 6 [file Table1.docx]

| Patient Number | Patient Name | Age | gender | Keloid Duration (Years) | Previous Treatment History | Family History | Atopic Status |
| --- | --- | --- | --- | --- | --- | --- | --- |
| 1 | Wang Ling | 33 | female | 6 | None | grandmother | None |
| 2 | Fan Lang | 28 | female | 15 | None | No | None |
| 3 | Cui Lixuan | 23 | female | 7 | None | No | None |
| 4 | Zhu Mengwen | 35 | female | 23 | None | No | None |

Supplementary Table 1. Patients information

| Sample ID | Sample name | Age | gender | Cell count | Median UMI | Median genes |
| --- | --- | --- | --- | --- | --- | --- |
| 240717031 | Inf1 | 33 | female | 27265 | 3948 | 1472 |
| 240717033 | Hyp1 | 33 | female | 23185 | 4613 | 1619 |
| 240807017 | Inf2 | 28 | female | 16949 | 5468 | 1794 |
| 240807018 | Hyp2 | 28 | female | 17230 | 4613 | 1640 |
| 240816044 | Inf3 | 23 | female | 19447 | 4047 | 1537 |
| 240816045 | Hyp3 | 23 | female | 21873 | 3306 | 1340 |
| 241011061 | Inf4 | 35 | female | 11658 | 5345 | 1872 |
| 241011060 | Hyp4 | 35 | female | 10547 | 4488 | 1686 |

Supplementary Table 2. Transcriptome sequencing sample information

| \| Cell Type \| \| --- \| | Inf | Hyp |
| --- | --- | --- | --- |
| Fibroblasts | 2570（3.90%） | 4136（6.59%） |
| Keratinocytes | 50111（76.00%） | 45304（72.20%） |
| Endothelial cells | 5153(7.81%) | 4043(6.44%) |
| Mast cells | 1083(1.64%) | 2266(3.61%) |
| Melanocytes | 1032(1.65%) | 1032(1.56%) |
| Mononuclear macrophages | 2482(3.76%) | 1316(2.1%) |
| Mural cells | 2044(3.10%) | 3122(4.98%) |
| Schwann cells | 569(0.86%) | 599(0.95%) |
| T and NK cells | 899(1.36) | 912(1.45%) |

Supplementary Table 3. **Total counts and proportions of different cell types in single-cell sequencing outcome**
